# The nature of methadone diversion in England: a Merseyside case study

**DOI:** 10.1186/1477-7517-9-3

**Published:** 2012-01-13

**Authors:** Paul Duffy, Helen Baldwin

**Affiliations:** 1 Criminal Justice System Manager, Centre for Public Health, Liverpool John Moores University, 2nd Floor, Henry Cotton Campus, 15-21 Webster Street, Liverpool, L3 2ET, UK; 2 Researcher in Substance Misuse, Centre for Public Health, Liverpool John Moores University, 2nd Floor, Henry Cotton Campus, 15-21 Webster Street, Liverpool, L3 2ET, UK

**Keywords:** Methadone, diversion, treatment, supervision

## Abstract

**Background:**

Methadone maintenance treatment (MMT) is a key element in treatment for opiate addiction; however concerns about the diversion of methadone remain. More current empirical data on methadone diversion are required. This research investigated the market for diverted methadone in Merseyside, UK, in order to provide a case study which can be transferred to other areas undertaking methadone maintenance treatment on a large scale.

**Methods:**

Questionnaires were completed (in interview format) with 886 past year users of methadone recruited both in and out of prescribing agencies. Topic areas covered included current prescribing, obtaining and providing methadone, reasons for using illicit methadone and other drug use.

**Results:**

Large proportions of participants had obtained illicit methadone for use in the past year with smaller proportions doing so in the past month. Proportions of participants buying and being given methadone were similar. Exchange of methadone primarily took place between friends and associates, with 'dealers' rarely involved. Gender, age, whether participant's methadone consumption was supervised and whether the aims of their treatment had been explained to them fully, influenced the extent to which participants were involved in diverting or using diverted methadone.

**Conclusion:**

Methadone diversion is widespread although drug users generally do not make use of illicit methadone regularly (every month). The degree of altruism involved in the exchange of methadone does not negate the potential role of this action in overdose or the possibility of criminal justice action against individuals. Treatment agencies need to emphasise these risks whilst ensuring that treatment aims are effectively shared with clients to ensure adherence to treatment.

## Background

### Evidence of Methadone Diversion

Whilst the effectiveness of the various models of methadone treatment has been established through international research [1-5], concerns have been expressed about the potential 'leakage' of methadone onto the 'black market' and the increased risk of harm that this entails [6-8]. The diversion of methadone has been implicated as a key contributing factor in fatal and non-fatal methadone poisonings [9-12]. The risk of street selling of methadone and its association with overdose have also been recognised by drug users being prescribed substitute medication [13].

Investigative studies in developed countries have examined the prevalence and nature of methadone diversion and illicit use [14-17]. Findings suggest methadone is used and sold illicitly at a high level relative to other prescription opioids, although the diversion of buprenorphine has also become widespread [18]. Illicit methadone use is associated with recent heroin use [14,19,20] and methadone is used illicitly in conjunction with other illegal substances, such as illicit benzodiazepines [21]. It is usually administered orally however studies conducted with drug users in England and Australia reveal injecting of oral solution [22-24].

Research findings also provide insight into how the illicit market for prescription methadone is generated. Illicit methadone is commonly used as self-medication for the management of withdrawal symptoms by opiate addicted individuals not engaged in treatment [20,25]. It is also obtained by clients in treatment looking to supplement their own methadone prescription or replace their prescription after failing to obtain it due to missed appointments or prescription pick-ups [21,25,26]. A key motive for selling prescribed methadone is to raise funds to buy other, preferred, drugs or to pay for private prescriptions which cannot be obtained as part of the free provision of care [27]. Common tactics used to obtain excess methadone for illicit sale include acquiring more than one prescription and obtaining a higher dosage than required [28]. The cost of methadone on the illicit market fluctuates according to supply and demand [27].

### Protocols for Prescribing and Dispensing Methadone

Prescribing and dispensing practices can facilitate or hinder the diversion of methadone; overly large dosages and 'take-home' prescriptions have been strongly associated with accelerated diversion [8,29]. Official guidelines for the prescribing and dispensing of methadone in the UK state that measures should be taken to prevent diversion, mainly implementing supervised consumption and prescribing methadone in its oral form [30,31]. However variations in prescribing protocols are apparent between UK regions and individual prescribers [32-34] and prescribing often deviates from guidelines [35]. Prescribing and dispensing practices also vary internationally, in terms of the doses of methadone prescribed [36,37] and the use of supervised consumption [38]. Historically, concerns about diversion have been a trigger for increased regulation [35].

### Methadone Diversion in Merseyside

Merseyside (population 1,353,400 in 2010 [39]) is a mixed rural and urban, affluent and deprived, county in the North West of England and it has been identified as a diverse area with relatively high problematic drug use in some parts and not in others [40]. The diversity of the area and its population make it good candidate for a study of this type and findings will be transferable to other areas undertaking methadone maintenance on a large scale. During the 1980s Merseyside saw a 'heroin epidemic' [41]. An inter-agency approach to tackling this major heroin problem using methadone maintenance therapy (MMT) alongside other harm reduction approaches successfully reduced the prevalence of illicit drug use and acquisitive crime, although methadone leakage became a concern [42]. Although models of drug treatment in the UK are becoming more 'recovery' focused, with greater focus on rehabilitation, recovery networks and reintegration [43], MMT remains a key element in the treatment for opiate addiction both nationally and in the region [44]. During the year 2009/10, 8,759 drug users accessed general practitioner or specialist prescribing in Merseyside (C. Gibbons, personal communication, February 23, 2011). However methadone diversion remains an issue, with drug users coming into contact with drugs workers in custody suites reporting illicit methadone use via national monitoring systems.

### Research Aims

Further empirical data on the magnitude and trends of the diversion of prescription opioids are needed to inform clinical decisions and risk management [38,45]. Little recent published work has investigated the diversion of methadone in England. This research aimed to examine the extent and nature of methadone diversion from the perspective of a large sample of opiate users in Merseyside. Findings will inform drug treatment practitioners and commissioners across the UK as to the mechanics of the market for illicit methadone, to enable them to consider best practice in addressing the issue of diversion with clients. There are difficulties transferring learning on this topic to other countries due to large variations in treatment delivery approach, drugs used and legislation but it is hoped that many aspects of the findings will be of use to practitioners and commissioners involved in the delivery of MMT in other countries with established treatment systems.

## Methodology

### Participants

Participants were recruited from 28 sites across Merseyside between November 2008 and September 2010. Sites included primary prescribing services (community drugs teams), agencies providing treatment specifically for offenders, service user forums and services providing accommodation for drug users. Recruitment approaches varied from site to site but generally advertising materials were placed in waiting rooms/communal areas or handed out by practitioners indicating when members of the research team would be in attendance. On the designated recruitment days the research team attended the service and approached individuals within communal areas or practitioners directed potential participants to them. Any individual over 18 and who had used methadone (licit or illicit) in the previous year was eligible.

### Materials

A 28 item questionnaire with a mixture of closed and open questions was developed after an examination of previous literature and with input from a number of drug treatment practitioners. Topics covered included current prescribing, obtaining and providing methadone, reasons for using illicit methadone and other drug use. No personal details aside from age and gender were collected to ensure confidentiality and reassure participants in an attempt to promote full disclosure. Before commencing the interview all participants were asked to confirm that they had not taken part in the interview previously.

### Procedure

Once participants had indicated their desire to take part in the study they received a full explanation of the project (verbal and written) including assurances around confidentiality and were asked to provide written consent for their participation. The questionnaire was completed by the researcher in an interview with the participants (lasting approximately 15 minutes). Participants received a travel voucher (worth £4) as reimbursement for their time.

### Analysis

All analysis was conducted using PASW Statistics 17.0. The Chi-square test for association was used for comparisons across categorical items (e.g. gender) and independent measures t-tests were applied for continuous data (e.g. age of participants).

### Ethics

The design and procedure of the project, including the questionnaires, were reviewed and approved by both the authors' university ethics committee and a National Health Service Research Ethics Committee.

## Results

### Sample Characteristics

A total of 886 participants were recruited aged between 18 and 64 years old (mean 38, sd 7.03). The majority of the sample were male (71%). These characteristics are broadly similar to national (England) figures for individuals engaged in treatment in 2009/10 (median age 33, 73% male) [46]. In 2009/10 the total number of individuals receiving General Practitioner or agency based prescribing in Merseyside was 7,392. The sample represents 12% of this population or 7% of the estimated opiate using population within the geography (latest available estimate for 2008/9) [47]. The majority of participants (86%) were prescribed methadone at the time of their interview, with all but 29 of the sample prescribed methadone within the past year. Among those participants currently prescribed methadone, 85% were receiving doses of 80 mg a day or less (28% of the sample were receiving less than 40 mg a day). Frequent prescription pick-ups were the norm with 72% of participants picking up daily and 22% several times a week.

### Reasons for using prescribed methadone

Participants reported a wide variety of reasons for obtaining a prescription, primarily 'to avoid withdrawal' (84% of participants), 'to aid them to achieve abstinence' (22%), 'to relieve unpleasant mental states e.g. anxiety' (18%), 'to stabilise their lifestyle' (11%) or 'to stop them committing crime' (8%). Regardless of the reasons stated, high levels of satisfaction were reported with prescribed methadone's ability to achieve this goal with only 5% of participants claiming that methadone was ineffective for their stated expectation.

### Supervision

More than half of the sample (53%), were not on supervised consumption at the point they were interviewed, so many were in a position to divert their methadone if that was their desire. Among those supervised, 60% said they would prefer not to be and gave a variety of reasons for this including embarrassment at taking methadone in the chemist, inconvenience and a desire for greater control over the time and location of use including splitting doses (half in the morning and half in the evening). A lack of satisfaction with supervision was significantly associated with clients feeling that the reasons why they were on supervision had not been explained to them fully (χ2 = 13.23, p < 0.05).

### Size of the market

60% of participants suggested that at some point in the past year they had obtained illicit methadone whilst 22% said they had done so in the past four weeks. This identifies the presence of a substantial market for diverted methadone. However, the discrepancy between current (4 wks) and previous (yr) usage, suggests that it is not a market that clients routinely use. Much lower proportions of clients reported providing methadone to others over the same time periods (14% past year, 5% past 4 wks). It may be that clients were less willing to discuss the provision of their methadone to others for fear that it would prejudice their future treatment despite reassurances to the contrary. Analysis supported this as there was a significant association between where clients were recruited and reports of providing methadone in the previous year (χ2 = 34.924, p < 0.001). A greater proportion of participants recruited outside of their prescribing agency reported providing their methadone to others than those recruited at the agency (28% compared to 11%).

**Figure 1:**
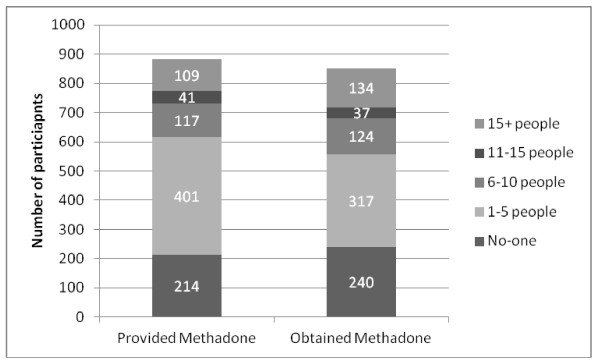
**Number of individuals participants knew who regularly provided or obtained methadone illegally**.

The size of the market is also indicated by reports from clients about the number of people they knew who regularly provided or obtained methadone illegally; 76% of participants knew at least one person who provided their methadone to others at least once a month whilst 72% knew at least one person who obtained illicit methadone at least once a month. For both obtaining and providing methadone more than three in ten participants said they knew more than five people who engaged in this activity at least once a month (Figure 1).

Questions about price revealed that 100 mg of methadone rarely cost more than £10 (only 3% of participants reported paying over this amount in the past year).

### The nature of the market

Most diversion of methadone was reported to take place between friends or associates. Very small proportions of participants undertook transactions with an individual they identified as a 'dealer' although where money was exchanged (to either buy or sell methadone) dealers were more commonly reported (however still in a small proportion of cases). When obtaining methadone the market would appear to be split equally into a cash based process (43% of participants had bought methadone in the past year) and a process where methadone, on the surface, is provided for free (44% of participants had been given methadone in the past year). Money was less likely to be involved when participants were providing their methadone to others with 13% of participants giving away their methadone in the past year compared to 5% who reported selling it. Participant's reticence to admit monetary gain from the process may in part explain the lower proportions of individuals reporting selling methadone (Table 1).

**Table 1:** Numbers of participants reporting diversion activity and who with

Diversion Activity	Time period	Number (%)	Who from/to	Number (%)
Been given methadone (n = 885)	Past Yr	387 (44)	Friend/Associate	346 (89)

			Partner	30 (8)

			Relative	9 (2)

			Doctor	2 (1)

			Dealer	10 (3)

			Other	8 (2)

	Past 4 wks	126 (14)	Friend/Associate	112 (88)

			Partner	10 (10)

			Relative	4 (4)

			Doctor	4 (3)

			Dealer	0

			Other	2 (2)

Bought Methadone (n = 884)	Past Yr	382 (43)	Friend/Associate	343 (90)

			Partner	3 (1)

			Relative	0

			Doctor	

			Dealer	19 (5)

			Other	14 (4)

	Past 4 wks	131 (15)	Friend/Associate	119 (91)

			Partner	0

			Relative	0

			Doctor	0

			Dealer	6(5)

			Other	3 (2)

Given away methadone (n = 854)	Past Yr	111 (13)	Friend/Associate	84 (76)

			Partner	30(27)

			Relative	4(4)

			Doctor	0

			Dealer	0

			Other	0

	Past 4 wks	35 (4)	Friend/Associate	24 (69)

			Partner	11(31)

			Relative	1(3)

			Doctor	0

			Dealer	0

			Other	0

Traded methadone (n = 855)	Past Yr	28 (3)	Friend/Associate	27(96)

			Partner	0

			Relative	0

			Doctor	0

			Dealer	1(4)

			Other	1(4)

	Past 4 wks	9 (1)	Friend/Associate	9(100)

			Partner	0

			Relative	0

			Doctor	0

			Dealer	0

			Other	1(11)

Sold methadone (n = 854)	Past Yr	46 (5)	Friend/Associate	43(93)

			Partner	1(2)

			Relative	0

			Doctor	0

			Dealer	0

			Other	3(7)

	Past 4 wks	14 (2)	Friend/Associate	14(100)

			Partner	0

			Relative	0

			Doctor	0

			Dealer	0

			Other	0

Clients who obtained methadone in the past year were significantly older than their counterparts who had not obtained methadone (t(884) = -2.436, p < 0.05). However there was no significant difference in the ages of clients who had or had not provided methadone to others (t(884) = 0.121, ns). The gender of participants was significantly associated with the provision of methadone in the past year (χ2 = 4.982, p < 0.05) but not with obtaining methadone (χ2 = 0.016, ns). One in ten female participants (10%) reported providing methadone to others in the past year compared to 16% of males. Participant's perceptions of the effectiveness of their prescribed methadone and their current prescribed dose levels were not significantly associated with obtaining or providing methadone either in the past year or past 4 weeks. Whether participants were currently supervised was significantly associated with obtaining (χ2 = 10.699, p < 0.005) and providing methadone (χ2 = 6.479, p < 0.05) in the past year (supervised participants more likely to have obtained methadone but less likely to have provided methadone) and providing methadone in the past four weeks (χ2 = 7.341, p < 0.01) (supervised participants less likely to report providing methadone in the past four weeks) but there was no association between supervision and obtaining methadone in the past 4 weeks (χ2 = 0.322, ns). Obtaining methadone in the past year (χ2 = 7.111, p < 0.01) and past 4 weeks (χ2 = 4.481, p < 0.05) were also significantly associated with participant's perceptions about the extent to which the aims of their treatment had been explained to them. In both cases clients who felt that the aims of their treatment had not been fully explained to them were more likely to report that they had obtained methadone illicitly (Table 2).

**Table 2:** Interaction of individual factors with diversion activity

Factor		Obtained methadone in past year	Obtained methadone in past 4 weeks	Provided Methadone in past year	Provided methadone in past 4 weeks
		**Number**	**Number**	**Number**	**Number**

Gender	Male (n = 631)	379	132	98	37

	Female (n = 255)	152	66	25*	4

Is methadone effective	Yes (n = 725)	410	149	88	37

	No (n = 40)	25	11	7	4

Dose Level	High (above 80 mg/day) (n = 113)	71	31	19	9

	Low (below 80 mg/day) (n = 650)	363	129	75	32

Currently Supervised	Yes (n = 362)	228	79	33	11

	No (n = 402)	206***	81	61*	30**

Aims of treatment fully explained	Yes (n = 652)	358	128	76	34

	No (n = 111)	76**	32*	18	7

*Significant at p < 0.05, **Significant at p < 0.01, ***Significant at p < 0.005

## Discussion

Rates of methadone diversion or use of diverted methadone seen in this study indicate the presence of a relatively large active 'black market' for methadone which a substantial proportion of participants had utilised but in general not regularly, evidenced by the differences between proportions of clients reporting obtaining illicit methadone in the past year compared to past four weeks. This suggests that there are specific circumstances under which this occurs. Due to different populations, national policies and time frames, comparisons of rates of use identified in this study with those from other countries are difficult. Among opiate users in New York studies have shown high rates of lifetime illicit methadone use (53% [48] and 72% [14]), and relatively high recent use (21.3% in the previous 6 months) [48]. Among a population of heroin users in Montreal, 42% had used illicit methadone in the previous 6 months [19]. In Dublin, 73% of opiate users presenting for treatment had used illicit methadone and 55% used at least once whilst in treatment [25]. In Australia among a sample presenting for drug treatment, 51% had used methadone illicitly in the year before presentation [24]. Comparisons with other geographies can also prove difficult due to the differing levels of 'treatment penetration'. Areas where access to treatment is relatively easy with short waiting times would be assumed to have less need for drug users to turn to the use of street drugs or to diverted methadone thereby minimising the market. Merseyside like many parts of the UK has good access to prescribing with short waiting times therefore it could be assumed this is not a major driver for the size of the diverted methadone market.

The reportedly low price of illicit methadone in Merseyside (as seen in other research [24]) suggests that there is a large enough supply in the market to keep this price low [49-51].

A large proportion of clients reported being prescribed under the optimal dose levels recommended in the UK guidelines on clinical management of drug use and dependence [30] which are suggested to be 60-120 mg a day. A phenomenon of under prescribing is not unique to the UK having been reported in a number of other countries [52,53]. There is a considerable body of evidence that higher doses are associated with more positive treatment outcomes [36,54-59] and doses over 100 mg of methadone have been associated with more positive outcomes in terms of tests for illicit opiates [60]. So the 'topping up' that has been seen in this study and in others [26] may be associated with the relatively low levels of prescribing reported by participants. However, high levels of satisfaction with the effectiveness of methadone in achieving participant's stated reasons for using methadone and a lack of association between perceptions of effectiveness or dose levels and diversion suggest that it is not a lack of efficacy of prescribed methadone that is a driver for the illicit market. This supports findings from other work [26] which indicated that adequacy of dose was not related to levels of illicit methadone use. The impact of prescribing higher doses of methadone in terms of clients' potential desire (and the desire of the current UK Government) to move towards abstinence as soon as possible also requires some consideration. Higher doses may lead to greater dependency on methadone and subsequent longer, more difficult periods of detoxification.

High proportions of clients on supervised consumption reporting dissatisfaction with this status raises some potential issues with disengagement from services, especially if reasons for not liking supervision are as emotive as embarrassment due to the locality of chemists or the lack of appropriate private consumption rooms [61] which are critical in clients satisfaction with supervised consumption [62]. Also within the framework of the current UK focus on recovery [43] with clients being reintegrated into mainstream society as quickly as is safe, factors such as inconvenience and a lack of control over prescribing have potential to disempower drug users seeking to make positive changes [63-65]. However, this must be balanced against the health risks posed by unregulated use of methadone in the community both to individuals already prescribed, those not engaging with services and non drug users [66-68]. Previous work has suggested that taking the opposite approach, starting unsupervised and moving to supervised when concerns around appropriate use become apparent, can lead to greater attrition [69]. Disempowerment and disengagement are also potential outcomes if clients feel that they are not a part of their treatment planning [44,70] and, in this study, participants who felt that they had not had the aims of their treatment explained were more likely to report 'topping up' their methadone prescription. In addition levels of satisfaction with being on supervised consumption were associated with the degree to which participants felt the reasons for this had been explained to them fully.

The desire for additional control over when and how methadone could be taken e.g. some in the morning and some in the afternoon, may be linked to participants anxiety about the ability of their methadone to hold them for the full 24 hrs [71] despite the fact that as long as dosing is correct the pharmacology of methadone should mean this presents no problem. This may link again though to the relatively low doses being prescribed, although as outlined most participants found that their methadone was effective for their purposes.

Supervised consumption was associated with lower levels of past year and past four week diversion implying that having this control in place is effective, despite doubts expressed by drug treatment agency key workers in Merseyside [27]. Findings support previous work indicating that supervised consumption can promote adherence to prescribed methadone use [72]. The high frequency of pick-ups reported should also mean that individuals do not have large quantities to pass on at any one time, unless they are stockpiling, and should help to guarantee adherence to treatment [72].

The methadone market does not appear to be commercially motivated with most diversion happening between friends or associates (not with 'dealers') and often with no money changing hands, suggesting a considerable altruistic element or the expectation of reciprocation in the future. It may also be indicative of a process of self medication among drugs users. Clients who are self titrating (only by small amounts at a time) will have small amounts of methadone left over which are then available to friends and associates. Despite this non commercial profile legal considerations remain. Individuals caught with someone else's prescription or caught passing their prescription to another person could face substantial criminal sanctions (methadone is controlled under Class A of the Misuse of Drugs Act 1971 with a potential sentence of up to life in prison for an offence of supply or seven years for possession). In reality the risk of detection may be relatively small and it is not clear that in all cases criminal justice agencies would apply the full measure of the law. In addition this level of risk may be little disincentive to individuals intent on doing so and who are likely to have an existing criminal record. Despite this it may be prudent for practitioners to emphasise this potential consequence of diversion along with clinical aspects e.g. overdose [9-12].

It is not clear why older participants should be more likely to report obtaining illicit methadone in the past year. It may be that these individuals are more likely to have received methadone treatment previously, in the past have had more reason to utilise the market for methadone [26] and know other drug users who have access to methadone when compared to younger clients who may not have been prescribed in the past. From the data collected for this study it is not clear why females should be less likely to provide methadone to others than males. It may reflect a number of factors including; greater risks perceived by this group in terms of the impact on dependants if they were caught providing methadone (and an associated fear of revealing diversion); a higher perceived need for treatment among this group (previous studies have suggested females are more likely to use street methadone possibly indicating an increased need for treatment [20] or greater levels of motivation for recovery. Unfortunately this is not an area that has been investigated in previous work on methadone diversion. It is a point that will need to be tackled in future research.

### Study Limitations

The possibility of underreporting particularly among participants recruited within prescribing agencies must be considered (as seen from findings where individuals recruited outside prescribing agencies reported higher levels of diversion and use of the diverted methadone). In particular clients who are diverting their methadone on a large scale are unlikely to agree to discuss this with a researcher who is asking about methadone diversion. Removal of this under reporting would only serve to increase the suggested size of the market which is already assessed as being considerable. The potential reticence of participants to discuss diversion with an independent researcher, who has no involvement with the delivery of treatment raises questions about the extent to which clients would report any diversion to clinicians. As practitioners have reported that objective measures to detect diversion (e.g. drug testing) are not effective [61] subjective judgements must be relied upon which are not reliable and if action is based on these it is likely to cause conflict.

Measures to ensure that individuals felt comfortable discussing a sensitive topic such as collecting the bare minimum of personal details may introduce the possibility that participants were interviewed twice. A small group of interviewers and the use of a filter question to determine prior completion should have minimised this issue.

This sample in this study was mostly of a group that have been engaged with treatment relatively recently, further investigation with opiate users not in contact with treatment is required to better understand the experiences of this group as potential consumers of diverted methadone. However, as a general rule a number of those clients engaged in treatment must at some point be the suppliers of methadone so their perception of individuals involved in the market can be considered accurate.

## Conclusions

Whilst there continues to be a considerable market for diverted methadone, opiate users would generally appear to make use of it relatively infrequently. Supervision would appear to be an appropriate measure for controlling diversion, however the potential effect of its long term imposition on a client's recovery journey should be considered especially as long term restrictions on take home doses have been shown to impede treatment engagement [73,74]. The market is characterised as much altruism as by commercial motivation but the health and criminal justice risks posed by diversion to individuals attempting to tackle their addiction remain considerable. Treatment agencies need to emphasise these risks whilst ensuring that treatment aims are effectively shared with clients to ensure adherence to treatment.

## Competing interests

The authors declare that they have no competing interests.

## Authors' contributions

PD conceived of the study, participated in its design, conducted the analysis and produced the final manuscript. HB led on the design and co-ordination of the study (including fieldwork), conducted and wrote the literature review. All authors have read and approved the final manuscript.
